# Empirical Estimation of Information Measures: A Literature Guide

**DOI:** 10.3390/e21080720

**Published:** 2019-07-24

**Authors:** Sergio Verdú

**Affiliations:** Independent Researcher, Princeton, NJ 08540, USA; verdu@informationtheory.org

**Keywords:** information measures, empirical estimators, entropy, relative entropy, mutual information, universal estimation

## Abstract

We give a brief survey of the literature on the empirical estimation of entropy, differential entropy, relative entropy, mutual information and related information measures. While those quantities are of central importance in information theory, universal algorithms for their estimation are increasingly important in data science, machine learning, biology, neuroscience, economics, language, and other experimental sciences.

## 1. Introduction

The edifice of information theory is built upon the foundation of three major information measures (e.g., [1]):

- **Entropy:**H(P) of a probability mass function *P* on a discrete set A:
  (1) $$\begin{array}{c} H\left(P\right)=\sum_{a\in A}P\left(a\right)\log\frac{1}{P(a)}. \end{array}$$
- **Relative Entropy:**D(P∥Q) of a pair of probability measures (P,Q) defined on the same measurable space (*P* and *Q* are known as the dominated and reference probability measures, respectively; X∼P indicates P[X∈B]=P(B), for any event *B*):
  (2) $$\begin{array}{c} D\left(P∥Q\right)=E\left[\log\frac{dP}{dQ}\left(X\right)\right],\;X∼P. \end{array}$$
- **Mutual Information:**I(X;Y) of a joint probability measure $P_{XY}$:
  (3) $$\begin{array}{c} I\left(X;Y\right)=D\left(P_{XY}∥P_{X}\times P_{Y}\right), \end{array}$$
  which specializes to I(X;X)=H(X) in the discrete case.

Using bits, or other information units, those nonnegative measures gauge the randomness of *P* (entropy); the dissimilarity between *P* and *Q* (relative entropy); and the statistical dependence of *X* and *Y* (mutual information). They attain their minimum value, zero, if and only if *P* is deterministic, P=Q, and *X* and *Y* are independent, respectively.

We would not be far off the mark if we were to define information theory as the study of the properties of those information measures, in particular, as they pertain to their role in the fundamental limits of various operational engineering problems such as the compression and transmission of data.

Often, when dealing with random processes with memory, the role of those information measures is supplanted by their asymptotic counterparts: entropy rate, relative entropy rate, and mutual information rate, defined as the asymptotic linear growth in *m* of the corresponding measure for a block of *m* random variables, e.g.,

(4) $$\begin{array}{c} H\left(X\right)=\lim_{m\to \infty }\frac{1}{m}H\left(X_{1},\ldots ,X_{m}\right). \end{array}$$

The importance of information measures transcends information theory. Indeed, since shortly after their inception, a wide variety of experimental sciences have found significant applications for entropy, mutual information and relative entropy. For example,

- *Ecology* [2];
- *Economics* [3,4];
- *Finance* [5];
- *Language* [6,7,8,9];
- *Machine learning* [10,11,12];
- *Molecular biology and genomics* [13,14,15];
- *Neuroscience* [16,17,18,19];
- *Psychology* [20,21];
- *Signal processing* [22]; and
- *Statistics* [23,24,25,26].

Frequently, in those applications, the need arises to estimate information measures empirically: data are generated under an unknown probability law, and we would like to design a machine learning algorithm that estimates the information measure, assuming that the random data are stationary and ergodic, so that time averages converge to statistical averages. In fact, much more is usually assumed, and, at this point in time, the state-of-the-art in the design and analysis of empirical estimators is much more advanced in the ideal case of independent identically distributed data.

Another application of such universal empirical estimators is to approximate (4) (or relative entropy rate or mutual information rate) when the distribution is known but the analytical computation of the limit is not feasible.

Although we limit this survey to entropy, relative entropy and mutual information, there are other information measures, distances between probability distributions, and statistical dependence measures whose empirical estimation has received considerable interest, such as Rényi entropy (including support size) and Rényi divergence [27], *f*-divergence [28], erasure entropy [29], total variation distance, Hellinger distance, $\chi^{2}$ distance (e.g., [30]), directed information [31], and lautum information [32].

## 2. Entropy: Memoryless Sources

**Figure 1:**
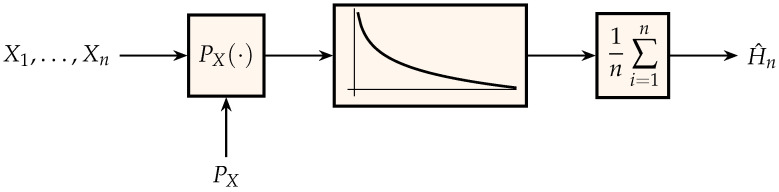
Generation of an estimate for entropy where the middle block is the function $\log\frac{1}{t}$, t∈(0,1].

The output estimate, ${\hat{H}}_{n}$, of the scheme in Figure 1 is *consistent*, i.e., it converges to the entropy, because of the law of large numbers. Unfortunately, it has no practical utility as an empirical estimator of entropy since it requires knowledge of $P_{X}$. Nevertheless, the scheme in Figure 1 suggests a two-pass algorithm for estimating the entropy of samples drawn independently from an unknown distribution by replacing the unknown $P_{X}$ in the left block with its empirical estimate ${\hat{P}}_{X}^{\left(n\right)}$ computed from the *n* observations $X^{n}=\left(X_{1},\ldots ,X_{n}\right)$:

(5) $$\begin{array}{c} {\hat{P}}_{X}^{\left(n\right)}\left(a\right)=\frac{1}{n}\sum_{i=1}^{n}1\left\{X_{i}=a\right\},\;a\in A. \end{array}$$

Alternatively, in view of Equation (1), once we have ${\hat{P}}_{X}^{\left(n\right)}$, we can just compute its entropy by means of

(6) $$\begin{array}{cc} {\hat{H}}^{\left(n\right)}\left(X^{n}\right) & =\sum_{a\in A}f\left({\hat{P}}_{X}^{\left(n\right)}\left(a\right)\right), \end{array}$$

(7) $$\begin{array}{cc} f(t) & =t\log\frac{1}{t}. \end{array}$$

The *plug-in* universal estimator of entropy in Equation (6) is a maximum-likelihood estimator, which converges almost surely, as n→∞, to H(X).

**Figure 2:**
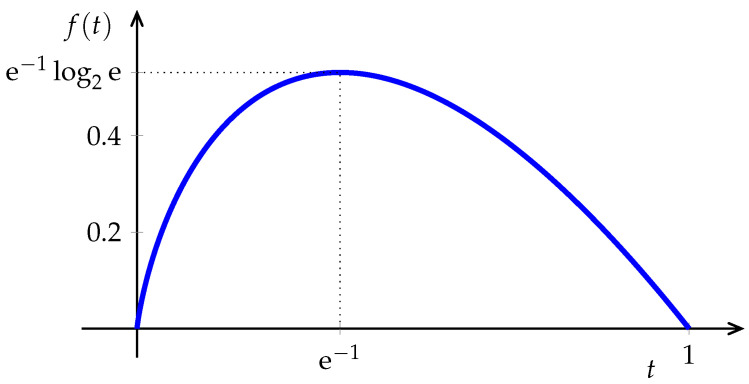
The function $t\log_{2}\frac{1}{t}$.

Because of the strict concavity of Equation (7) and $E\left[{\hat{P}}_{X}^{\left(n\right)}\left(a\right)\right]=P_{X}\left(a\right)$, for any *n* the estimate in Equation (6) is underbiased,

(8) $$\begin{array}{c} E\left[{\hat{H}}^{\left(n\right)}\left(X^{n}\right)\right]<H\left(X\right), \end{array}$$

except in the trivial deterministic case. Although, as n→∞, the bias (which is equal to the relative entropy between the empirical distribution and the unknown $P_{X}$) vanishes, in applications with large alphabets the bias may be appreciable unless *n* is exceedingly large. The reason is that the innocuous looking function in Figure 2 has an infinite right-derivative at 0, thus, for distributions with many infrequent symbols, errors in the empirical estimates of their probabilities translate into large errors in their contributions to the sum in Equation (6). This challenge has received considerable attention, particularly in recent years. In addition to standard statistical *bias-reduction* techniques (e.g., [33,34,35,36,37]) and *shrinkage* techniques [38], one way to ameliorate this issue is to distort the function f(·) in Equation (6) by replacing the initial portion with a polynomial (see, e.g., [39,40,41,42]). Incidentally, we note that the idea of classifying the symbols in the alphabet into two categories (those with large enough probability whose frequency is plugged in Equation (6) directly, and those trouble-making infrequent ones that need to be dealt with separately) goes back to the work of Dobrushin [43] in 1958, which deals with the infrequent symbols by averaging the logarithms of their interoccurrence times. This approach is applied in [44] (see also [45]) to obtain, with 2n observations, the same performance that the plug-in estimator would obtain with $n\sqrt{\log n}$ observations, not only for the estimation of entropy, but a large class of information measures.

Demanding consistency and low bias from the empirical estimator is desirable but not enough to obtain a useful estimator. How can we estimate the sample size *n* that a given algorithm will require in order to output a reliable estimate? The quality of the empirical estimator is usually judged by its worst case mean-square error over all possible $P_{X}$ (or a subset if some prior knowledge is available). A lower bound on the worst case mean-square error incurred by the plug-in estimator is obtained in [41,46], which also shows (see also [47]) that the plug-in method requires nlogn samples to achieve the same performance that a minimax estimator can achieve with *n* samples, so there is plenty of motivation to search for optimal or near-optimal computationally feasible solutions instead of using the plug-in method.

Paninski [39] gave a non-constructive proof of the existence of consistent estimators of entropy when the number of observations is less than the order of the alphabet size. Applications where the number of observations is much smaller than the alphabet size are increasingly common in modern machine learning; in that domain, it is futile to try to have an accurate empirical distribution. However, even if the number of observations grows only as $O\left(\frac{\left|A\right|}{\log|A|}\right)$, with a “constant” that, naturally, depends on the sought-after estimation accuracy, Valiant and Valiant [48] proposed a computationally intensive linear-programming based algorithm that estimates entropy accurately. Moreover, Valiant and Valiant [49] showed a converse result stating the impossibility of lower sample complexity in the regime in which the number of observations is not too large and the bias, rather than stochastic fluctuations, is responsible for most of the inaccuracy. The bias of the absolute error incurred by the linear programming algorithm of Valiant and Valiant [48] is upper bounded as the square root of the bias $\frac{\left|A\right|}{n\log|A|}$ achieved both by a plug-in to a distorted target function [40,41] and by the linear programming type algorithm proposed in [50].

Entropy estimators that are linear on the *fingerprint* or *profile* (histogram of the histogram) of the observed sample were studied, among others, by Valiant and Valiant [47], Jiao et al. [40], Wu and Yang [41]. Nonlinear processing of the fingerprint may be more efficient, as pointed out in [51], which shows the optimality (even beyond entropy estimation) of algorithms based on finding the distribution that maximizes the likelihood of the observed fingerprint, also known as the *profile maximum likelihood* method. Efficient algorithms for such nontrivial optimization are proposed in [52,53], the latter of which also considers the more general setting of Markov sources.

Less studied is the Bayesian approach to empirical entropy estimation, which improves accuracy by incorporating prior information about the unknown probability mass function (see, e.g., the work of Wolpert and Wolf [54] and Jiao et al. [40], who showed essentially the same nonasymptotic worst-case performance as the maximum likelihood estimate).

The behavior of the plug-in estimator of entropy when the discrete distribution has infinite support (e.g., in applications where unbounded waiting times are observed) is studied in [55,56]. Unless some restriction is placed on the possible set of probability mass functions, Antos and Kontoyiannis [56] showed that for any sequence of estimators, the worst-case convergence rate can be arbitrarily slow. A universal non-asymptotic upper bound on the variance of the plug-in estimator is also found in [56].

## 3. Entropy: Sources with Memory

We turn to the empirical estimation of the entropy rate in Equation (4) from a sample path of the finite alphabet stationary ergodic process X. In principle, for any integer *m*, we can estimate the empirical joint distribution

(9) $$\begin{array}{c} {\hat{P}}_{X^{m}}^{\left(n\right)}\left(a^{m}\right)=\frac{1}{n}\sum_{i=1}^{n-m+1}1\left\{\left(X_{i},\ldots X_{i+m-1}\right)=a^{m}\right\},\;a^{m}\in A^{m}, \end{array}$$

and then apply a plug-in approach or any of the methods surveyed in Section 2 to estimate $\frac{1}{m}H\left(X^{m}\right)$. We can then increment *m* until the estimate stabilizes to near its limit. However, except for toy examples, this approach is not computationally feasible because of the exponential dependence of the effective alphabet size on *m*, which dictates astronomical values of *n* to get reliable empirical estimates of the joint distribution.

Claude Shannon [6,57] made the first forays in the empirical estimation of the entropy rate of an English text, not by a machine learning algorithm but also by a human, who arrived at an estimate based not only on the text itself but on the knowledge of the syntax and lexicon of the language, in addition to any information useful in guessing the next letter based on the previous text. This is based on the fact that, if the entropy is finite, then the entropy rate can be expressed as the limit of the conditional entropy of the next symbol given the past:

(10) $$\begin{array}{c} H\left(X\right)=\lim_{m\to \infty }H\left(X_{m}|X^{m-1}\right). \end{array}$$

This line of research was continued by Cover and King [58], who came up with a convergent estimate based on sequential gambling on the next letter of the text.

Any optimal (i.e., achieving the entropy rate) universal data compression algorithm can be used as an estimator of the entropy rate by simply measuring the length of the output of the compressor. However, Jiao et al. [59] (see also [60]) gave evidence that insisting on compression incurs in higher sample complexity than entropy estimation: optimal universal compression (of memoryless sources) cannot be accomplished with |A| samples, while $O\left(\frac{\left|A\right|}{\log|A|}\right)$ suffices for entropy estimation. Under mixing conditions, Han et al. [61] showed that the sample complexity for Markov chains scales with the state space cardinality |A| as $O\left(\frac{{\left|A\right|}^{2}}{\log|A|}\right)$. The finite sample-size behavior of plug-in estimators for the entropy rate of unknown Markov chains is analyzed in [62] along with a corresponding algorithm for the estimation of Rényi entropy rate.

Even though empirical estimation of entropy rate has smaller sample complexity than universal compression at the Shannon limit, most successful algorithms for empirical estimation of entropy rate are inspired by universal data compressors. Notable exceptions include Kaltchenko’s nearest-neighbor entropy rate estimators [63,64,65].

In the general context of stationary ergodic sources, Ziv and Merhav [66] proposed using the number of Lempel–Ziv (LZ) parsing phrases times its logarithm, normalized by sequence length, as an estimate of the entropy rate. Grassberger [67] proposed an alternative family of consistent estimators for the entropy rate of stationary ergodic sources, which is suggested by a result of Wyner and Ziv [68], Ornstein and Weiss [69] that states that, for an arbitrarily chosen time $t_{0}$, the length $L_{n}$ of the shortest string occurring after $t_{0}$ that has no match as a substring occurring in $\left\{t_{0}-n,\ldots ,t_{0}\right\}$ behaves as

(11) $$\begin{array}{c} \frac{L_{n}}{\log n}\to \frac{1}{H(X)},\;a.s. \end{array}$$

Consistency was shown in increasing generality by Shields [70], Kontoyiannis and Suhov [71] and Kontoyiannis et al. [72]. Improved versions of matching-based estimators are proposed in [73].

An efficient method for universal entropy estimation based on block sorting (Burrows–Wheeler transform, BWT) is proposed in [74], and shown to achieve almost sure convergence to entropy rate, along with an analysis of its convergence rate for finite-alphabet finite-memory sources. The principle followed in [74] is to revert to the estimation of the entropy of memoryless sources, and therefore the quality of its estimates can be improved by importing the recent advances outlined in Section 2.

At the expense of increased software complexity over LZ compression, universal compressors based on arithmetic coding and sequential modeling algorithms such as context-tree-weighting (CTW) [75] and prediction by partial match (PPM) [76] achieve faster convergence to the entropy rate. These modeling algorithms compute an estimate of the probability of the next symbol given the past. Therefore, they can be readily adopted to estimate the conditional entropies in Equation (10). A comparison of empirical entropy estimators based on CTW and on matching [73] shows the practical superiority of CTW-based empirical entropy estimation. The related problem of empirical estimation of *erasure entropy* [29] is studied in [77] where a bilateral version of CTW is proposed.

## 4. Differential Entropy: Memoryless Sources

The *differential entropy* of a probability density function $p_{X}$ on the real line (or $R^{d}$) was introduced by Shannon [57] (and independently by Wiener [78]):

(12) $$\begin{array}{c} h\left(X\right)=-\int p_{X}\left(t\right)\log p_{X}\left(t\right)\;dt. \end{array}$$

Although not as fundamental as entropy, mutual information and relative entropy, the empirical estimation of differential entropy has also received considerable attention for memoryless sources. For example, a normality test can be based on whether the empirical differential entropy attains a value close to $\frac{1}{2}\log\left(2\pi e\;\sigma_{X}^{2}\right)$. A survey of the state of the art in 2009 of empirical estimation of the differential entropy of continuous random variables and vectors can be found in [79].

The scheme in Figure 1 can be readily adapted to estimate differential entropy by letting the left-block use an estimate of the probability density function. This approach is followed in [80,81,82,83,84]. In particular, Györfi and Van der Meulen [81] advocated using half of the samples as inputs to the scheme in Figure 1, and the other half to estimate the probability density function; input samples which correspond to very small values of the estimated probability density function are discarded.

Naturally, the most popular approach is to use a plug-in method where the density in Equation (12) is replaced by an estimate based on a histogram, or a kernel approximation (e.g., [80,85,86]). Note that the integrand in Equation (12) is also the function in Figure 2, except that now its domain extends to the whole positive real line. As argued in Section 2, in view of the infinite derivative at zero, it is to be expected that estimation inaccuracies will arise from improbable intervals. Restricting the possible density governing the data to satisfy a sufficiently large Lipschitz constraint enabled Han et al. [87] to show matching achievability and converse results showing that the square root of the minimax mean square error behaves as

$${\left(\frac{1}{n\log n}\right)}^{\frac{s}{s+d}}+\frac{1}{\sqrt{n}}$$

where s∈(0,2] is a smoothness parameter that governs the Lipschitz constraint, and *d* is the dimension of the observation vectors. As in the case of (discrete) entropy, Han et al. [87] showed that, without distorting the function in Figure 2 near the origin, plug-in methods are doomed to be strictly suboptimal.

Kozachenko and Leonenko [85] proposed the nearest neighbor estimator

(13) $$\begin{array}{c} {\hat{h}}^{\left(n\right)}\left(X^{n}\right)=\frac{1}{n}\sum_{i=1}^{n}\log\left(n\min_{j\neq i}\left|X_{i}-X_{j}\right|\right)+c \end{array}$$

where *c* is a constant. A truncated version of this estimate is shown in [88] to achieve a mean-square error of $O\left(\frac{1}{n}\right)$. More generally, *k*-nearest-neighbor methods for the empirical estimation of differential entropy have received considerable attention. A simple *k*-nearest-neighbor estimate is given by

(14) $$\begin{array}{c} {\hat{h}}_{k}^{\left(n\right)}\left(X^{n}\right)=\frac{1}{n}\sum_{i=1}^{n-k}\log\left(\frac{n}{m}\left(X^{\left(i+k\right)}-X^{\left(i\right)}\right)\right) \end{array}$$

where $X^{\left(1\right)}\leq X^{\left(2\right)}\leq \ldots \leq X^{\left(n\right)}$ denote the sorted version of $X^{n}$. If in addition to n→∞, *k* is allowed to grow, $\sqrt{n}\left({\hat{h}}_{k}^{\left(n\right)}\left(X^{n}\right)-h\left(X\right)\right)$ is shown in [89] to be asymptotically normal with zero mean and variance $\mathrm{Var}(\log p_{X}\left(X\right))$ under the assumption that $f_{X}$ is bounded and bounded away from zero on its support. Other nearest-neighbor methods for the empirical estimation of differential entropy are proposed and analyzed in [90,91,92,93,94]. An upper bound on the minimax mean-square error attained by nearest-neighbor estimators of differential entropy is established in [95].

Estimation of other nonlinear functionals of probability density functions, beyond differential entropy, has received considerable attention in the statistics literature (see, e.g, [96]).

## 5. Relative Entropy: Memoryless Sources

**Figure 3:**
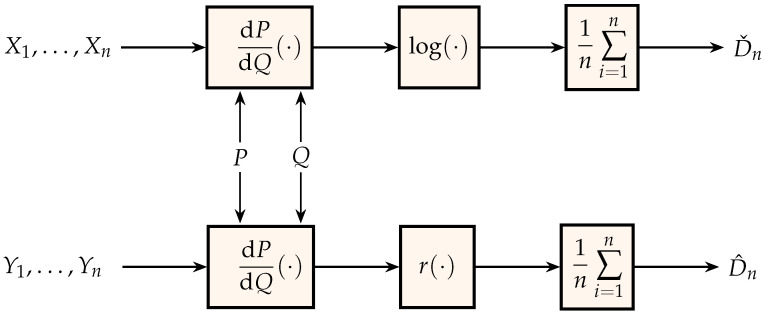
Generation of estimates for relative entropy.

In the same spirit as Figure 1, Figure 3 shows two estimators of the relative entropy $D(P_{X}∥P_{Y})$ between the probability measures that generate the independent identically distributed sequences $X_{1},X_{2},\ldots$ and $Y_{1},Y_{2},\ldots$. The middle block in the bottom estimator is the nonnegative function

(15) $$\begin{array}{c} r(t)=(1-t)\log e+t\;\log t. \end{array}$$

To see the rationale for this, note that, in addition to Equation (2), we can express relative entropy as

(16) $$\begin{array}{c} D\left(P∥Q\right)=E\left[r\left(\frac{dP}{dQ}\left(Y\right)\right)\right],\;Y∼Q. \end{array}$$

Empirical estimation requires a two-pass algorithm in which the first pass estimates the unknown density $\frac{dP}{dQ}\left(a\right),a\in A$ (see, e.g., [97,98]).

**Finite alphabet.** In the discrete case, we can base a relative entropy estimator on the decomposition
  (17) $$\begin{array}{c} D\left(P_{X}∥Q_{X}\right)=-H\left(X\right)+E\left[{ı}_{Q_{X}}\left(X\right)\right],\;X∼P_{X} \end{array}$$
  with ${ı}_{Q}\left(a\right)=\log\frac{1}{Q(a)}$. Therefore, once an empirical estimator of entropy is available, the task is to design an algorithm for estimating the cross term. To that end, it is beneficial to regularize the probability estimate for those symbols that are infrequent under the reference measure. Various regularization and bias-reduction strategies are proposed in [99,100], leading to consistent estimators.
  In the memoryless case, several of the algorithms reviewed in Section 2 for entropy estimation (e.g., [40,47]) find natural generalizations for the estimation of relative entropy. As for entropy estimation, the straightforward ratio of empirical counts can be used in the plug-in approach if |A| is negligible with respect to the number of observations. Otherwise, sample complexity can be lowered by a logarithmic factor by distorting the plug-in function; an estimator is proposed in [101], which is optimal in the minimax mean-square sense when the likelihood ratio is upper bounded by a constant that may depend on |A|, although the algorithm can operate without prior knowledge of either the upper bound or |A|. Another nice feature of that algorithm is that it can be modified to estimate other distance measures such as $\chi^{2}$-divergence and Hellinger distance. The asymptotic (in the alphabet size) minimax mean-square error is analyzed in [102] (see also [101]) when the likelihood ratio is bounded by a function of the alphabet size, and the number of observations is also allowed to grow with |A|.
**Continuous alphabet**. By the relative entropy data processing theorem,
  (18) $$\begin{array}{c} D\left(P_{X}∥Q_{X}\right)\geq D\left(P_{\varphi (X)}∥Q_{\varphi (Y)}\right) \end{array}$$
  where φ:A→B, and B is arbitrary. If φ is injective, then the equality in Equation (18) holds. If we choose B to be a finite set, then an empirical estimate of the lower bound in Equation (18) can be obtained using one of the methods to estimate the relative entropy for finite alphabets. It is to be expected that keeping the number of bins |B| small results in lower complexity and coarser bounds; on the other hand, allowing |B| to grow too large incurs in unreliable estimates for the probabilities of infrequent values of φ(a),a∈A. Of course, for a given B, the slackness in Equation (18) will depend on the choice of φ. An interesting option propounded in [103] is to let φ be such that $Q_{X}\left(\varphi^{-1}\left(b\right)\right)$ is independent of b∈B, in which case
  (19) $$\begin{array}{c} D\left(P_{\varphi (X)}∥Q_{\varphi (Y)}\right)=\log\left|B\right|-H\left(P_{\varphi (X)}\right). \end{array}$$
  Naturally, we cannot instrument such a function since we do not know $Q_{X}$, but we can get a fairly good approximation by letting φ depend on the observations obtained under the reference measure, such that each bin contains the same number of $Y_{i}$ samples. Various strongly consistent algorithms based on data-dependent partitions are proposed in [79,103].
  For multidimensional densities, relative entropy estimation via *k*-nearest-neighbor distances [104] is more attractive than the data-dependent partition methods. This has been extended to the estimation of Rényi divergence in [105]. Earlier, Hero et al. [106] considered the estimation of Rényi divergence when one of the measures is known, using minimum spanning trees.
  As shown in [107], it is possible to design consistent empirical relative entropy estimators based on non-consistent density estimates.
  The empirical estimation of the minimum relative entropy between the unknown probability measure that generates an observed independent sequence and a given exponential family is considered in [108] with a local likelihood modeling algorithm.
  *M*-estimators for the empirical estimation of *f*-divergence (according to Equation (16), *r*-divergence with r(t) in Equation (15) is the relative entropy)
  (20) $$\begin{array}{c} D_{f}\left(P∥Q\right)=E\left[f\left(\frac{dP}{dQ}\left(Y\right)\right)\right],\;Y∼Q; \end{array}$$
  where *f* is a convex function with f(1)=0 are proposed in [109] using the Fenchel–Lagrange dual variational representation of $D_{f}\left(P∥Q\right)$ [110]. A *k*-nearest neighbor estimator of $D_{f}\left(P∥Q\right)$ is proposed and analyzed in [111].
  A recent open-source toolbox for the empirical estimation of relative entropy (as well as many other information measures) for analog random variables can be found in [112]. Software estimating mutual information in independent component analysis can be found in [113]. Experimental results contrasting various methods can be found in [114].

## 6. Relative Entropy: Discrete Sources with Memory

Dropping the assumption that the unknown sources are independent presents considerable new challenges for the empirical estimation of relative entropy rate, which have not yet been addressed in the realm of analog data. In the finite-alphabet case, a paradigmatic application is the quantitative measure of the “statistical similarity” between long texts.

A useful starting point is to leverage Equation (17) and separate the task into entropy estimation and the estimation of the cross term (average of information under the reference measure with respect to the other measure). Universal data compression techniques have inspired a variety of algorithms for the empirical estimation of the cross term starting with [66], which is proposed using a cross Lempel–Ziv-like parsing of a sequence with respect to another. Consistency is shown in [66] under the assumption that both sources are Markov and independent. Text classification using the algorithm in [66] was studied by Pereira Coutinho and Figueiredo [115]. A heuristic application of Lempel–Ziv compression is proposed in [9] motivated by the fact that, according to Equation (17) and basic results in data compression, the relative entropy rate is, approximately, the penalty in length when instead of using an optimum compressor tuned to $P_{X}$, we use a compressor tuned to $Q_{X}$. This suggests training a universal compression algorithm with the text generated under $Q_{X}$ and measure its compression length under the other text. Instead of relying on Lempel–Ziv compression, Cai et al. [99] used the Burrows–Wheeler transform (BWT), which transfers the redundancy in the data due to memory into redundancy due to non-uniformity of the marginals of the piecewise stationary asymptotically independent outputs. The main algorithmic challenge is to segment the outputs adaptively in order to determine the points at which the statistics switch. In the algorithm in [99], the Burrows–Wheeler transform is applied to the concatenated texts, and the segmentation is done with respect to the reference text, so as to produce an estimate of the cross term in Equation (17). Consistency of the estimator is shown in [99] along with an analysis of its convergence rate for finite-alphabet finite-memory sources, and an experimental application to texts: the *Bible* written in various languages and novels of various authors. Further experimentation with the *Universal Declaration of Human Rights* written in various languages and a mammalian DNA evolutionary tree are reported in [116] (see also [117]) using data compression algorithms. These and other experimental results led Kaltchenko [118] to conjecture that relative entropy is a more powerful discriminator than other measures of statistical distance.

## 7. Mutual Information: Memoryless Sources

Discussing specific data in soccer (influence of home/away in scoring goals), the spread of wildfires, and neuroscience, Brillinger [119] made a case for the benefit of empirical estimates of mutual information in various data science applications over other more established statistical dependence-testing tools. In particular, a natural application is testing the independence of the components in *independent component analysis* (e.g., [113,120]).

If both random variables are discrete, then we can leverage empirical estimators of entropy in order to estimate mutual information since, in that case,

(21) $$\begin{array}{c} I(X;Y)=H(X)+H(Y)-H(X,Y). \end{array}$$

A similar approach can be followed when both random variables are analog by using algorithms to estimate the differential entropy (Section 4). A word of caution is that, in some applications with weakly dependent random variables, the error in the entropy estimator may be non-negligible with respect to the mutual information. Although the plug-in estimator of entropy is underbiased, using it in conjunction with Equation (21) yields estimates of mutual information that may be positively or negatively biased depending on the joint distribution [39]. Although largely unexplored (see [121,122] for recent work), the empirical estimation of mutual information between discrete *X* and analog *Y* is also of interest in a number of applications; for example, suppose that X∈{red,green,blue} and *Y* is an intracellular voltage trace from visual cortex neurons.

In the domain of memoryless analog sources, generally preferable to differential-entropy based methods are estimation algorithms that exploit the definition of mutual information as a relative entropy between the joint probability measure and the product of its marginals (Equation (3)), thereby opening the possibility of using the approaches taken in Section 5. The upper scheme in Figure 3 has been used for the estimation of the mutual information of analog random variables in [82,123,124]. Note that those algorithms have not yet been extended to deal with sources with memory. For those cases, a pragmatic approach is advocated in [79] where multimedia data are pre-processed by a standard lossy compression front-end (such as JPEG or MPEG), which projects on a multiresolution basis producing essentially memoryless streams of analog data. Then, the algorithms for mutual information estimation of memoryless sources can be applied to those streams. Naturally, the projections in off-the-shelf standard software have very low complexity and they leave residual correlations among the streams, which result in both data rate inefficiencies and estimation inaccuracies.

A widely used empirical estimator of I(X;Y) for real-valued random variables is proposed in [125] by simply quantizing the real-line and using a plug-in estimator for relative entropy. The variance of the estimate is closely approximated by the variance of the information density, $\log\frac{dP_{XY}}{dP_{X}\times P_{Y}}\left(X,Y\right)$, normalized by the number of observations. The highly influential estimator proposed by Fraser and Swinney [126] goes one step further by adapting the quantization size to the data, leading to a more systematic treatment of data-dependent partitions (not necessarily products of scalar quantizers) in [127]. Even greater adaptivity is beneficial by having equally-populated partitions (see [128] and its supplemental material). Those approaches are studied more systematically in the more general context of relative entropy estimation [79,103].

*k*-Nearest-neighbor estimators of mutual information are studied in [79,104,120,129].

*Directed (mutual) information* [31] defined for two time series as

(22) $$\begin{array}{c} I\left(X^{n}\to Y^{n}\right)=\sum_{i=1}^{n}I\left(X^{i};Y_{i}|Y^{i-1}\right) \end{array}$$

is important in the fundamental limits of channels with memory with feedback, and has been proposed for quantifying the elusive notion of causality. Empirical estimators of directed information for finite alphabet processes are proposed in [130,131], the latter of which shows an optimal rate of convergence of $O(n^{-1/2})$ and adapts the estimator to testing for causality.
